# Treatment of Tooth Pulps

**Published:** 1869-12

**Authors:** J. G. Willis

**Affiliations:** Cincinnati, Ohio


### TREATMENT OF TOOTH PULPS.

BY J. G. WILLIS, M. D., CINCINNATI, OHIO.

No complication attending the decay of teeth has given the profession more trouble than the exposure of their pulps. Its consideration has added largely to the literature of our Dental journals, and has afforded the widest field for the display of purely scientific ability of those members of the profession, who have seen fit to make public their views, and the practice formed thereon.

The gravity of this complication can not be easily overestimated, and notwithstanding the extensive consideration which the subject has elicited, through the journals and societies in all sections of the country, there is no settled course of procedure recognized by the profession, as being preferable to all others. The methods of treatment when the same result is desired to be obtained, are as diametrically opposite as the results themselves—the life or death of the pulp.

Heretofore the question has been how best to save a *tooth* after a pulp was exposed, and the earliest means adopted for that purpose frequently, if not always, resulted in the death of the pulp and ultimately, if relied on solely in the loss of the tooth itself, from ulceration, abscess and internal decay. The propriety of destroying exposed pulps under any circumstances, I will not at this time consider, except incidentally. As a complete exposition of the subject would consume more of my time than at present I can spare.

But the best practice—and the best because the most successful—in cases of exposed pulps, when their death is decided to be imperative, has not yet been agreed upon by the profession, and therefore the practice in such cases is widely variant, and results as widely. That the profession may have another method of practice in such cases, from which to select; I will, as briefly as the subject will admit, give mine in detail, and the reason therefor. I use arsenic for the devitalization of the pulp—the usual formula, viz: arsenic, creosote and morphine—before applying to the pulp I clean out the cavity of decay, as thoroughly as possible, and with the excavator locate the opening into the pulp cavity, and enlarge it as much as the patient will allow. The removal of decayed and discolored dentine allows a better view to be had of the cavity, and enlarging the opening into the pulp cavity insures a more thorough application of the destructive agent. I make the preparation very *thin* with creosote before using, and saturate a pledget of cotton, and press it directly upon the exposed pulp; this pressure usually causes some pain, and the pain is a sure indication that you have made the application at the right point; therefore, if no pain is complained of, I continue to move the cotton around until the outcry of the patient notifies me that I have hit the spot. I continue a moderate pressure for a minute or so to give the preparation time to insinuate itself between the pulp and the walls of its containing cavity. The fluidity of the preparation renders this a matter of no difficulty whatever, and the death of the pulp is rendered much more certain, than where the preparation is used thicker. I rarely have to make the second application, and never, except in cases in which the exposure is small and can not be increased by reason of pain. I then, with another pledget of cotton, absorb the surplus creosote, and stop the cavity with *wax*, and I endeavor to press the wax in the direction of the exposed pulp, so as to be sure that the latter is not moved from its contact with the opening into the pulp cavity. This usually also causes some pain, which assures me everything is all right. I direct the patients to allow the wax to remain four hours, and four hours *only*, when they are to remove it and the cotton, and return next day.

After considerable experimenting, in which my object was to find the shortest possible time required to kill a pulp, I have settled upon four hours, and with a reasonable amount of exposure, will not fail once in fifty times to produce the desired result. Again, I do not think it proper to hermetically seal the cavity of decay, for the gases and fluids—the products of decomposition of the pulp—must find exit at some point, and if prevented from escaping through the crown, will be forced through the apical foramin, and irritation, congestion and inflammation of the investing membrane of the root will be the result, and if left sufficiently long, abscess will be the

*inevitable* result. Above all things, then, I caution all to never hermetically seal up a cavity, in which there is an agent destructive to the pulp.

Here it will be proper for me to say that if I could always do as I choose, I should extract the pulp mechanically; but as people will not consent to that operation, we have to select another. At the next sitting of the patient I enlarge the opening, and very generally without pain, and expose thoroughly the root canals. If I find some tenderness in the extreme end of the roots, I am not at all sorry, for by that I shall be assured that the effects of the preparation have not gone beyond the tooth, but are confined within its cavity. Frequently blood will escape from the canals; when this is the case, I regard the operation as in the same stage, as though the pulp had been mechanically extracted, and the object to be accomplished the same, viz: the healing of the divided pulp and the closure of the foramin. I seldom use the barbed nerve extractors, but when I have used one and safely removed it from the canal, I thank my stars that it did not break, and if I had then always thrown it away, I should have been saved great trouble from its subsequent breaking. They are bound to break sooner or later, and therefore should not be used once *too* often. I am exceedingly careful not to force an instrument through the apex of a root, as frequent failures result from this cause alone. And I never use a drill in a root; as from the frequent deviations of canals from straight lines, drills are often driven through the root against the alveolus, and this accident makes a failure inevitable.

I have seen three teeth, the extraction of which was made necessary from this cause. In one of them cotton, and in another, gold projected beyond the tooth, whi.e the third could not be got into a condition to fill, and when removed the reason was plainly apparent. These accidents all happened in the practice of as good Dentists as the country affords, and should be a warning to all when they attempt to use drills in such places.

After removing all the *debris* of the nerve I dry out the canals as thoroughly as possible with cotton, drawn out into a fine thread, slightly twisted, and carefully forced into the canals. I then saturate a similar thread of cotton with creosote, and lightly force into the root, leaving the end to project into the cavity, that it may be easily removed. Close the cavity with wax and direct the patient to call the next day. On the next visit I remove the cotton from the roots, again explore them thoroughly and make another application of creosote, as before, always being careful not to force creosote through the root. My object being to close up the tubuli and destroy any septic matter in the canals. The divided pulp will heal readily and entirely close the foramen and no fear need be apprehended of any fluids finding their way into the canal, whether plugged or not. Many Dentists labor under the delusion that the object of plugging the root canals is to prevent the accumulation of fluids in them; if there should be any fluid of any description, whatever, which would drain into the canal, if not plugged, the preventing of that drain would insure peri-cementitis, and perhaps loss of the tooth. At the next sitting of the patient, after removing cotton and thoroughly cleansing the canals, I force a very small thread of cotton, saturated with creosote, into the root as far as I can, absorb the excess of creosote, and plug the root canal, or not as is most desirable, if small, I just plug their mouths, if larger, I generally plug them through their whole extent.

If a tooth is not to be plugged as soon as it is in a condition to be *safely* plugged, the crown cavity must be sealed up with something, to prevent the access of fluids of the mouth to the root canals. I use Hill's stopping for this purpose. I often plug the roots and then wait for a day or two to observe results; if no trouble supervenes, I plug the crown cavity, with complete confidence that there is perfect immunity from danger. A tooth is made sore sometimes by forcing threads of cotton into the canals too tightly, thereby preventing any drain which may come from dead tissue ; this can only be done with safety, after all foreign matter is removed from the root, or is converted into an indestructible compound by action of creosote. Whether a tooth is in a condition to be plugged or not, may be determined by plugging the crown *only*, with Hill's stopping. If there be any discharge it will soon manifest itself by uneasiness in the tooth when the stopping should be removed and creosote again applied. Many Dentists charge upon arsenic, all the soreness that follows its application to an exposed pulp ; but they forget that the same degree of soreness may be produced in a tooth with pulp already dead, by plugging the crown tightly, and the same result occurs in both cases from the same cause, with which the arsenic has nothing to do, except to produce the condition upon which decomposition depends. The above practice has been so thoroughly satisfactory in my hands that I feel warranted in assuring all who will follow it, that they need not lose a tooth treated in accordance with it, unless complicated by themselves. I have treated about fifty teeth with exposed pulps in this manner, during the last year, and have not had a complaint from one of them, and without a failure in a single instance. A ratio of success that can not but recommend the practice to all. I sincerely hope that this article may be the means of shedding light, and that by reason of it, some teeth may be saved which would otherwise be lost.

